# Exploring the Relationship Between Paternalistic Leadership, Teacher Commitment, and Job Satisfaction in Chinese Schools

**DOI:** 10.3389/fpsyg.2020.01481

**Published:** 2020-07-08

**Authors:** Xiao Shi, Zeyuan Yu, Xin Zheng

**Affiliations:** ^1^Faculty of Education, Southwest University, Chongqing, China; ^2^International College of Chongqing University of Posts and Telecommunications, Chongqing, China; ^3^Center for Studies of Education and Psychology of Ethnic Minorities in Southwest China, Southwest University, Chongqing, China

**Keywords:** paternalistic leadership, trust in the principal, job satisfaction, commitment to students, teacher commitment, Chinese contexts

## Abstract

Paternalistic leadership (PL) is prevalent in organizations in East Asia, but few studies have examined its potential effects in school contexts. This study explored the relationship between PL, trust in the principal, and teachers’ satisfaction and commitment to students, with a focus on the mediating role of trust in the principal in Chinese schools. Using a quantitative method, the study investigated 408 primary schoolteachers in mainland China. The results showed that the three dimensions of PL had different effects on teachers’ job satisfaction, trust in the principal, and commitment to students. Moral leadership had positive effects, while authoritarian leadership had negative effects on teachers’ job satisfaction and commitment to students. Meanwhile, trust in the principal played a mediating role of authoritarian and moral leadership on teachers’ job satisfaction and commitment to students. Finally, implications and suggestions are discussed for leadership practices in Chinese schools and those in similar cultures.

## Introduction

In the past several decades, leadership has played a key role in influencing student learning and school improvement (Day et al., 2011; Hallinger, 2011; Leithwood et al., 2017). The educational leadership field has been dominated mainly by models such as instructional leadership and transformational leadership, which were developed mainly in Anglo-American contexts (Day et al., 2011; Hallinger, 2011). However, since the 2000s, researchers have consistently argued that successful leadership practices can hardly escape from cultural contexts (Hallinger, 2011; Leithwood et al., 2017; Walker and Qian, 2018b). Therefore, emerging studies have focused on different leadership practices across different cultures, to develop a more international, comparative, and contextually bounded scholarship (Mertkan et al., 2017). Among these studies focusing on non-Western contexts, paternalistic leadership (PL) has captured much attention, as it is a prevalent leadership style but often ignored in research on educational leadership (Jackson, 2016; Bedi, 2019).

PL is widespread in organizations such as firms and schools in China (Farh and Cheng, 2000; Aycan, 2006). Chinese culture is always described as respecting authority, being collectivistic, and pursuing morality (Bush and Qiang, 2000; Tian and Sanchez, 2017), which highlights the importance of hierarchical systems based on morality, kindness, and deference to authority (Aycan, 2006; Bedi, 2019). Even though the concept of PL was originally described in Chinese firms, scholars have noted or examined its existence not only in East Asia but also in Latin America and the Middle East areas such as Mexico and Turkey (Aycan, 2006; Pellegrini and Scandura, 2008; Hiller et al., 2019). For example, in the Global Leadership and Organizational Behavior Effectiveness (GLOBE) project, Mansur et al. (2017) found that 22 of the 59 societies endorsed some variant of PL behaviors. As Aycan (2006) argued, paternalism is most likely to occur in cultures characterized by collectivism, high power distance, and high affectivity. In addition, although the term paternalism is associated with negative connotations in the West, numerous studies conducted in non-Western contexts have shown that such leadership is still prevalent and effective in many business cultures (Farh et al., 2008; Pellegrini and Scandura, 2008; Bedi, 2019).

In terms of current PL research, two research gaps are noted. First, the results of PL on employees’ outcomes are still inconsistent (Pellegrini and Scandura, 2008; Bedi, 2019). For example, some scholars have found that the three dimensions of PL each have certain effect outcomes, i.e., authoritarian leadership has negative effects on employees’ outcomes, whereas moral leadership and benevolent leadership has positive effects on employees outcomes (e.g., Wu et al., 2012); however, other studies have shown either the opposite effects or insignificant results (Erben and Guneser, 2008; Schaubroeck et al., 2017; Tian and Sanchez, 2017; Wang and Guan, 2018). Also, an increasing number of studies that focus on PL are based on management and leadership literature (Pellegrini and Scandura, 2008; Bedi, 2019), but few studies have been conducted in school contexts. PL may be a prevalent leadership style in schools (Cheng et al., 2002; Farh et al., 2008; Walker and Qian, 2018a), yet it is still not clear how PL can benefit teachers and school improvement. To fill these gaps in the literature, the current study attempts to explore the relationship between PL and teacher-related outcomes. In terms of the outcomes, the study selects job satisfaction and teacher commitment, which are attitudinal outcomes of teachers’ professional development (Evans, 2011). Teachers’ job satisfaction and commitment have been frequently considered as strong indicators for student academic achievement and school improvement (Nir, 2002; Park, 2005).

Furthermore, the study proposed that trust in the principal would mediate the relationship between PL and teacher satisfaction and commitment for the following two reasons. First, the study follows the framework that school leaders influence students and teacher developments through multiple paths, one of which is the emotional path (Hallinger, 2011; Leithwood et al., 2017). According to this theory, principals’ behaviors may influence teachers’ work attitudes and performance indirectly through their interactions and quality of relationship with teachers (Leithwood et al., 2017). Second, Chinese cultural context emphasizes social relationships (Farh and Cheng, 2000; Aycan, 2006; Pellegrini et al., 2010) and PL is regarded as relationship oriented (Chen et al., 2014; Zheng et al., 2020). In school contexts, trust in the principal denotes the quality of principal–teacher interactions (Bryk and Schneider, 2002; Tschannen-Moran, 2014). Trust indicates the quality of social relationships, which is a key factor for successful leadership practices (Day et al., 2011; Yin and Zheng, 2018). Previous studies have argued that trust may act as a mediator between leadership practices and teachers’ outcomes (Li et al., 2015; Yin and Zheng, 2018). In a very recent meta-analysis of PL, Bedi (2019) suggests that “future researchers should further explore the mediating role of employee trust in the relationships between PL and employee outcomes” (p. 33). Thus, the current study further explores the mediating role of trust in the principal.

## Literature Review and Hypothesis

### PL and Its Dimensions

The definition of PL proposed by Farh and Cheng (2000) has been most widely used, which defines PL as “a style that combines strong discipline and authority with fatherly benevolence and moral integrity couched in a personalistic atmosphere” (p. 94). Current empirical studies have shown two lines of research on PL. First, PL can be considered as a single construct (Uhl-Bien et al., 1990; Pellegrini and Scandura, 2008). For example, Uhl-Bien et al. (1990) study in Japan found that paternalism was positively and significantly related to career investments, high-quality leader-member exchange (LMX) relations, and employees’ job satisfaction. In educational context, Song (2016) found that principal’s PL was correlated with teachers’ teaching efficacy. The second line of research adopts the triad model of PL. Researchers have argued that PL is a multidimensional model that is usually composed of three dimensions: authoritarianism, benevolence and morality (Farh and Cheng, 2000; Aycan, 2006). For example, Chen et al. (2014) found that benevolence and morality leadership are positively associated with both in-role and extra-role performance, whereas authoritarian leadership is negatively related to employee performance.

In this study, we adopted the triad model of PL. According to Farh and Cheng (2000), authoritarianism refers to leader behaviors that assert absolute authority and control over the subordinates and demand unquestionable obedience from them. Subordinates are expected to show their dependence and compliance to the leader. Benevolence refers to leadership behaviors that show individualized, holistic concern for subordinates’ professional, personal, and familial well-being. In return, subordinates will feel grateful and indebted to pay back to the leader. Morality refers to leadership behaviors that demonstrate personal virtue, self-discipline, unselfishness, and a leading role model, which lead to the subordinates’ respect for and identification with the leader.

In terms of the measurement of PL, Cheng et al. (2000) developed a scale, which consist of three distinct components: authoritarian leadership, benevolent leadership, and moral leadership. Each type was measured with items using a six-point Likert scale ranging from 1 (strongly disagree) to 6 (strongly agree). Participants rated their supervisors’ *shi-en* (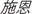), *li-wei* (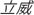), and *shu-de* (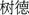) behaviors. The scale showed good internal reliability and construct validity (Cheng et al., 2000, 2004) and it was one of the most accepted and widely used measurements of PL (Mansur et al., 2017; Bedi, 2019). Cheng et al. (2014) revised the scales in their recent research, and selected and revised five items for each of the three dimensions. The new revised scale consists of 15 items, which captures the essence of authoritarian, benevolence, and moral leadership components in contemporary contexts (Cheng et al., 2014). It was applied in the present study.

### The Cultural and Chinese Context of PL

PL is not only culturally bounded but also contextually bounded (Pellegrini and Scandura, 2008; Mansur et al., 2017). Certain cultural and contextual factors have led us to conduct the current study.

Leadership practices have been found to vary widely across cultures and deeply depend on the cultural context (Farh and Cheng, 2000; Hallinger, 2011; Wu et al., 2012). As Aycan (2008) noted, leadership, especially PL, generally cannot be examined without considering its cultural context. According to Hofstede et al. (2010), Chinese culture is labeled as having a high-power distance and being collectivistic at the national level. A high power distance indicates a rigorous hierarchical relationship between principals and teachers. The principal’s authority is naturally accepted, and teachers are accustomed to showing compliance with their principals. Also, in collectivistic societies, social relationships matter for the operation of leadership practices, as in China (Walker and Qian, 2018b). The principals’ care about their teachers’ professional and personal lives is highlighted in school contexts (Yin and Zheng, 2018). Meanwhile, an emphasis on obligation and loyalty fits well within collectivistic societies. These culture rules necessitate principals’ benevolent behavior toward teachers. In return, teachers will reciprocate their gratitude and repayment toward principals.

In addition, Chinese cultures have the traditions of respecting for authority and maintaining harmonious relationship (Bush and Qiang, 2000; Walker and Qian, 2018b). The tradition of respect for authority widely exists in school contexts (Bush and Qiang, 2000). To some extent, a school is like a family where the principal is always portrayed as the father who has authority and teachers are akin to that of the children who need guidance and protection (Bush and Qiang, 2000; Walker and Qian, 2018a). Harmonious social relationship is highly appreciated in Chinese culture (Qian et al., 2017), which is considered as an element of successful leadership practices (Qian et al., 2017; Walker and Qian, 2018a). School principals needs to establish and maintain good relationships with teachers and they would care about teachers’ professional and personal lives (Yin and Zheng, 2018; Walker and Qian, 2018a).

The current Chinese contexts elicit some reasons for the study. First, Chinese schools are characterized by a “*principal responsibility system*,” in which principals have large power in personnel management, finance management, student enrollment, and decision making (Zhu et al., 2014). As Wong (2003) observed, successful principals appear to be a combination of “manager” and “clan leader.” On the one hand, principals use their power and authority to manage the school smoothly; on the other hand, they act as parents, or family members to buffer distractions from outside to establish a good relationship with teachers (Yin and Zheng, 2018; Walker and Qian, 2018b).

Second, the Ministry of Education in China launched the first *Professional Standards for Compulsory Education School Principals*, which proposed six professional responsibilities for principals: setting school development plan, creating a nurturing culture, optimizing internal management, engaging in curriculum and instruction, facilitating teacher development, and adjusting to external environment (Zheng et al., 2019). The standards indicate that principals nowadays should take more responsibilities and deal with multiple tasks in situations. Principals who have been accustomed to leading based on clear orders assigned from the top now are suggested to pay more attention to teachers’ development and establish good relationships with them (Walker and Qian, 2018b; Zheng et al., 2019). To be noted, the standards emphasize much on principals’ morality, which means that “a principal should be ethically moral, be impartial and honest, and care for teachers and students” (Liu, 2015, p. 9). In this changing circumstance, principals are facing increasingly more complex tasks and taking full responsibility in all aspects of school affairs, similar to a father figure of a big family. It may be necessary for principals to use multiple strategies that combine authoritarian, benevolent, and moral behaviors when they face different problems in different situations.

Considering the cultural and contextual features in China and the characteristics of PL, we propose that the three dimensions of PL may bring about organizational outcomes. Furthermore, trust may close the power distance and facilitate principal–teacher interactions.

### PL and Its Consequences

This study selected teachers’ job satisfaction and commitment to students as outcomes, which are two important indicators of teacher development. Teacher’s job satisfaction is defined as “teachers’ affective reactions to their work or to their teaching role” (Skaalvik and Skaalvik, 2011, p. 1030). Teacher commitment is defined as “teachers’ psychological attachment to the teaching profession, professional associations and school, colleagues, parents and students” (Lee et al., 2011, p. 821). As a multidimensional construct, teacher commitment is classified into three types: commitment to the teaching profession, to the students, and to the school (Firestone and Pennell, 1993; Razak et al., 2010; Lee et al., 2011). Only the teacher commitment to students was taken into account in this study, which is defined as teacher’s “devotion to and responsibility for student learning and behavior” (Park, 2005, p. 464). Researchers have continuously found that teacher commitment to students highly correlates with student achievement (Park, 2005; Lee et al., 2011; Frelin and Fransson, 2017).

Leadership behaviors have been consistently recognized as significant factors influencing teachers’ job satisfaction and commitment (Bogler, 2001; Ross and Gray, 2006). Previous studies have found that transformational leadership, servant leadership, and distributed leadership have positive effects on teachers’ job satisfaction and commitment (Nguni et al., 2006; Leithwood and Beatty, 2008). A few organizational studies have explored the relationship between PL and employee job satisfaction and commitment, most of which have examined the differential impacts of the three dimensions of PL on organizational variables (Niu et al., 2009; Zhang et al., 2015).

Authoritarian leadership functions as a typical command-based leadership style (Wu et al., 2012), which emphasizes maintaining strong authority over subordinates. Some studies have demonstrated that authoritarian leadership is not appreciated by subordinates and is negatively correlated with commitment to the team, satisfaction, job performance, intention to stay, loyalty toward leaders, trust in supervisors, and organizational commitment (Cheng et al., 2002; Farh et al., 2008). For example, Zheng et al. (2020) found that authoritarian leadership has negative effects on teachers’ commitment to the profession and commitment to the organization. However, some recent studies have revealed that in some situations, authoritarianism may be positive (Tian and Sanchez, 2017; Wang and Guan, 2018). For example, authoritarian leaders set an expectation of high standards and punish employees for poor performance (Tian and Sanchez, 2017), which can help employees gain a better understanding of what they should and should not do within the group (Wang and Guan, 2018).

Therefore, we propose that authoritarian leadership in school contexts will bring some negative influences on teachers’ job satisfaction and commitment to students. Our first hypothesis is as follows:

H1: Authoritarian leadership is negatively correlated with teachers’ job satisfaction and commitment to students.

Benevolent leaders are similar to kind fathers in that they display genuine concern for their subordinates’ job-related and personal well-being, which in turn makes the subordinates feel grateful (Farh and Cheng, 2000; Aycan, 2006). Benevolence leadership is likely to arouse a subordinate’s positive reciprocity because it shows goodwill toward the subordinate’s well-being (Wu et al., 2012). The leader’s benevolent behaviors should have a positive effect on subordinates’ job satisfaction and commitment, which may induce better performance as a return of the leaders’ care and concern. In current studies, benevolent behaviors have been found to be positively related to satisfaction with the team’s leader, commitment to the team, affective and continuance commitment, deference to supervisor, work motivation, in-role and extra-role performance, and job satisfaction (Cheng et al., 2002; Erben and Guneser, 2008; Niu et al., 2009; Chen et al., 2014; Bedi, 2019). For example, Bedi (2019) found that benevolent leadership was positively associated to subordinates’ satisfaction, affective commitment, and continuance commitment. Therefore, the second hypothesis is as follows:

H2: Benevolent leadership is positively associated with teachers’ job satisfaction and commitment to students.

Moral leadership highlights behaviors such as performing personal virtues and self-discipline, being selfless and setting an example. It can be considered as the most significant qualities for a Chinese leader (Wu et al., 2012). Subordinates who live in a society ruled by people rather than by laws and regulations always hold a high expectation for the leader’s moral behavior (Cheng et al., 2004). Subordinates tend to identify with a leader’s values and imitate a leader’s exemplary behaviors as a response to the leader’s moral behavior (Wu et al., 2012); thus, their job performance will be affected by the leader (Wu et al., 2012). Several empirical studies have demonstrated the positive relationship between moral leadership and subordinates’ affective and continuance commitment, satisfaction with the leader, commitment to the organization/team, and job satisfaction (Cheng et al., 2002; Farh et al., 2008; Afsar, 2014; Bedi, 2019). For example, Afsar (2014) found that moral leadership could enhance teacher’s affective and continuance organizational commitment. Therefore, a third hypothesis is as follows:

H3: Moral leadership is positively correlated with teachers’ job satisfaction and commitment to students.

### The Role of Trust in the Principal

The maintenance of interpersonal relationships (*guan xi*) is seen as a fundamental element of successful organizations in China (Wong, 2003; Yin and Zheng, 2018). Thus, trust is critical because it indicates the quality of interpersonal relationships between staff (Bryk and Schneider, 2002; Van Maele et al., 2014). Trust is defined as “the willingness to be vulnerable to another party based on the confidence that the other party is benevolent, honest, open, reliable, and competent” (Tschannen-Moran, 2014, p. 29). Faculty trust in schools is classified into three types: trust in the principal, trust in colleagues, and trust in clients (Tschannen-Moran, 2014). The current study focuses on the principal–teacher relationship; thus, trust in the principal is taken into account.

The relationship between leadership practices and trust in the principal has been explored in some studies. For example, Yin and Zheng (2018) found that leadership practices could significantly enhance teachers’ trust in the principal. Tschannen-Moran and Gareis (2015) found positive relationships between trust in the principal and instructional leadership. A few studies have investigated the relationship between PL and trust in supervisors. For example, Wu et al. (2012) indicated that moral and benevolent leadership are positively associated with trust in supervisors, while authoritarian leadership is negatively correlated with trust in supervisors. Chen et al. (2014) found that benevolent and moral leadership are positively related to affective trust in a leader, while authoritarian leadership is negatively related to affective trust in leader. Rawat and Lyndon (2016) found that benevolent and moral leadership enhanced trust in supervisor, while authoritarian leadership had no significant effects on trust in supervisor. Hiller et al. (2019) meta-analysis concluded that benevolent and moral leadership would enhance trust in principal, while authoritarian leadership would impair trust in principal. Based on prior studies, the fourth hypothesis of this study is proposed as follows:

H4: The three dimensions of PL are significantly correlated with trust in the principal.

Numerous organizational studies have demonstrated the positive relationship between trust in leaders and employees’ job satisfaction. In general, employees who trust in their leaders feel more satisfied with their jobs than employees who do not trust their leaders. In a meta-analytic study conducted by Dirks and Ferrin (2002), the authors summarized that trust in leaders was associated with employee attitudinal outcomes, particularly organizational commitment and job satisfaction. Skaalvik and Skaalvik (2011) found that positive social relationships with colleagues, parents, and school leaders were related to teachers’ job satisfaction. Van Maele and Van Houtte (2012) revealed a positive relationship between four types of teacher trust dimensions and job satisfaction.

A few studies have explored the relationship between trust in leaders and commitment. For example, Dirks and Ferrin (2002) research showed that higher level of trust in leadership were associated with higher levels of job satisfaction, higher organizational commitment, and lower intention of quitting. Miao et al. (2013) found that participative leadership triggers a higher level of trust in supervisors and leads to subordinates’ higher levels of organizational commitment. Lee et al. (2011) indicated that trust in colleagues was significantly and positively related to teachers’ commitment to students. Based on prior studies, the sixth hypothesis of this study is proposed:

H5: Trust in the principal is positively correlated with teachers’ job satisfaction and commitment to students.

Leadership studies have proposed that there is an indirect rather than direct relationship between leaders and employees (Leithwood et al., 2017), which has been confirmed by accumulated empirical research. For example, Chen et al. (2014) revealed that affective trust mediates the relationship between both benevolence and morality PL and employee performance but does not mediate the relationship between authoritarianism and employee performance. Wu et al. (2012) found that trust in supervisors completely mediates the relationship between supervisors’ moral and authoritarian leadership and subordinates’ in-role/ex-role performance, whereas trust in supervisors does not mediate the relationship between supervisors’ benevolent leadership and subordinates’ in-role/ex-role performance.

Trust plays a critical role in leader–teacher interactions because it can narrow the relational gaps between leaders and teachers (Van Maele et al., 2014). A number of current studies on Chinese principalship have revealed that principals do not frequently interact with individual teachers, and that they are inclined to influence teachers indirectly through redesigning school structure and establishing good relationships (Walker and Qian, 2018b; Zheng et al., 2019). This has led some researchers to explore the mediated effects of trust relationships between leadership practices and teachers’ performance. For example, Li et al. (2015) found that trust in the principal played as a mediator between principal leadership and teacher professional learning in Hong Kong primary schools. Therefore, the results mentioned above may imply that trust in the principal plays a mediating role in the relationship between PL and teacher outcomes. Thus, the sixth hypothesis of this study is proposed as follows:

H6: Trust in the principal significantly will mediate the relationship between three dimensions of PL behavior and teachers’ job satisfaction and commitment to students.

## Materials and Methods

### Participants

A total of 408 elementary schoolteachers from two southern provinces in China participated in this study. Teachers in Chinese schools are required to participate in professional development programs in local universities or teacher training colleges. Using a convenient sample, the researchers randomly asked the teachers to complete a questionnaire voluntarily when they joined the program in a local university and a teacher training college. They also filled the informed consent form that is approved by the authors’ University Survey Research Ethics Committee. The questionnaires and informed consent forms were administered by the first author. The sample consisted of 88 males (21.6%), 319 females (78.4%), and 1 missing value. Among them, 178 (43.6%) teachers taught Chinese language, 125 (30.6%) were mathematics teachers, 102 (25.0%) taught other subjects (e.g., English, science, and music), and 3 teachers did not report their subject. In terms of teaching experience, 102 (26.2%) had taught for 7 years or less, 96 (23.5%) had taught for 8–15 years, 104 (25.5%) had taught for 16–23 years, and 98 teachers (24.0%) had taught for 24 years or more; 8 teachers (1.9%) did not report their teaching age. The sample included 94 (23.0%) rural schoolteachers and 314 (77.0%) urban or suburban schoolteachers.

### Measures

A questionnaire with four scales, namely, the Paternalistic Leadership Scale (PLS), the Trust in the Principal Scale (TiPS), the Teacher Job Satisfaction Scale (TJSS), and the Teacher Commitment to Students Scale (TCSS), was used in this study. The scales are presented in the section “Appendix.”

The 15-item PLS adapted from Cheng et al. (2014) contains three subscales: Authoritarian Leadership (AL, five items), Benevolent Leadership (BL, five items), and Moral Leadership (ML, five items). The teachers rated each item on a six-point Likert scale ranging from “not at all” to “frequently.”

The five-item TiPS adapted from Louis et al. (2010) was used to assess teachers’ perceived trust in the principal. An example item is “In general, I believe my principal’s motives and intentions are good.” The TiPS was rated by teachers on a five-point Likert scale ranging from “strongly disagree” to “strongly agree.”

The TJSS consisting of five items was developed by Ho and Au (2006). An example item is, “My conditions of being a teacher are excellent.” The teachers rated these items on a five-point Likert scale ranging from “strongly disagree” to “strongly agree.”

The five-item TCSS adapted from Lee et al. (2011) was used to assess teachers’ commitment to students. An example is “it is my responsibility to ensure good social relations among my students.” The teachers were asked to rate each item on a five-point Likert scale ranging from “strongly disagree” to “strongly agree.”

All the four scales, PLS, TiPS, TJSS, and TCSS, have been used and validated in Chinese contexts (Lee et al., 2011; Cheng et al., 2014; Yin and Zheng, 2018). TiPS, TJSS, and TCSS were originally designed in English. A translation and back translation approach was conducted independently by two of the authors. Then, the authors invited five elementary schoolteachers to fill the questionnaire to ensure that the translation was clear to frontline teachers.

### Data Analysis

The data were analyzed using SPSS 19.0 and Mplus 7.0. First, a confirmatory factor analysis (CFA) was used to test construct validity of the scales. Second, the descriptive statistics and correlations were calculated by SPSS. Then, the structure equation modeling (SEM) and mediation analysis were conducted using Mplus. Several indices were used to indicate the robustness of fit for the CFA and SEM analyses, namely, the Chi-square statistic (χ^2^), the root mean square error of approximation (RMSEA), the Tucker–Lewis index (TLI), and the comparative fit index (CFI). As Hu and Bentler (1999) suggested, the cutoffs for the study are CFI > 0.96, TLI > 0.96, and RMSEA < 0.1 for indicating an acceptable data fit. For the mediation analysis, bootstrap was used to detect indirect effect (Hayes, 2009).

### Reliability and Construct Validity of the Scales

The reliability and construct validity of the four scales were examined. The results showed that all six factors had acceptable reliability coefficients, and their Cronbach’s alpha coefficients ranged from.68 to.89 (see Table 1). For the PLS, the three-factor structure of PL showed a good data fit (χ^2^ = 363.17, *df* = 87, *p* < 0.01, RMSEA = 0.088, CFI = 0.98, and TLI = 0.98), with factor loadings ranging from.41 to.97. TiPS also showed a good data fit (χ^2^ = 17.53, *df* = 5, *p* < 0.01, RMSEA = 0.078, CFI = 0.99, and TLI = 0.99). For the TJSS, the results showed an excellent data fit (χ^2^ = 12.78, *df* = 5, *p* < 0.05, RMSEA = 0.061, CFI = 0.99, and TLI = 0.99). The TCSS showed an acceptable model fit (χ^2^ = 18.36, *df* = 5, *p* < 0.01, RMSEA = 0.080, CFI = 0.99, and TLI = 0.99).

**TABLE 1:** Descriptive statistics, Cronbach’s α, and correlation matrix.

	**1**	**2**	**3**	**4**	**5**	**6**
1. AL	–					
2. BL	−0.29**	–				
3. ML	−0.32**	0.77**	–			
4. TIP	−0.35**	0.62**	0.66**	–		
5. JS	−0.48**	0.52**	0.60**	0.55**	–	
6. CS	−0.30**	0.49**	0.62**	57**	0.59**	–
*M*	2.76	5.12	5.57	4.72	4.41	4.84
*SD*	1.24	0.92	0.76	0.54	0.65	0.37
Cronbach’s *alpha*	0.76	0.79	0.89	0.86	0.68	0.87

**p < 0.01. AL = authoritarianism; BL = benevolence; ML = morality; TIP = trust in the principal; JS = job satisfaction; and CS = teacher commitment to students.

## Results

### Descriptive Statistics and Correlations

Table 1 presents the descriptive statistics for all of the factors. Among the three factors of PL, authoritarian leadership scored 2.76 (*SD* = 1.24) and morality (*M* = 5.57, *SD* = 0.76) scored higher than benevolence (*M* = 5.12, *SD* = 0.92). The mean score of job satisfaction was 4.41 (*SD* = 0.65), which was lower than that of teacher commitment to students (*M* = 4.84, *SD* = 0.37). Trust in the principal scored 4.72 (*SD* = 0.54). Table 1 displays the correlation matrix of the six factors, and all of the correlations are significant. As shown, authoritarian leadership was negatively correlated with job satisfaction and commitment to students; thus, H1 was supported. Benevolent and moral leadership were positively correlated with job satisfaction and commitment to students; thus, H2 and H3 were supported. Trust in the principal was negatively correlated with authoritarian leadership, and was positively associated with benevolent leadership, moral leadership, job satisfaction, and commitment to students. Therefore, H4 and H5 were supported.

### Structure Equation Modeling Results

**FIGURE 1:**
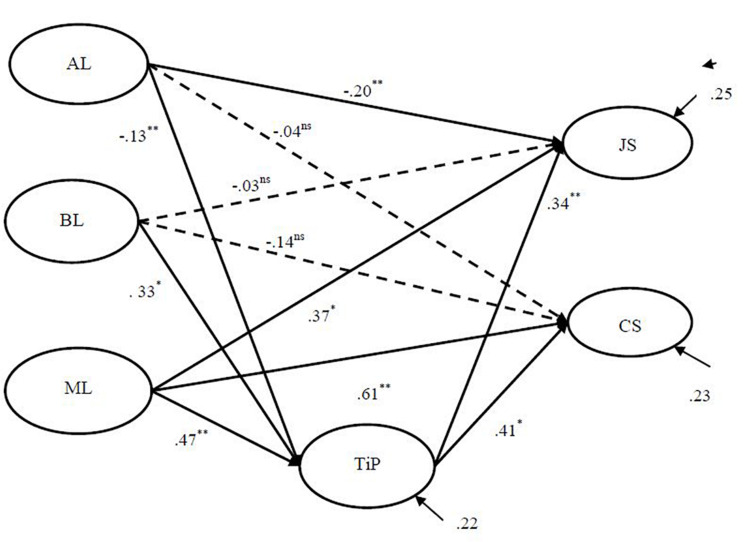
Mediating effect of job satisfaction and trust in the principal on the effects of PL on teachers’ job satisfaction and commitment to students. *Note*: ^∗∗^*p* < 0.01, ^ *^p < 0.05, and ns = not significant. Dotted lines indicate non-significant paths.

An SEM model was used to test the relationship between PL, job satisfaction, teacher commitment to students, and trust in the principal. The results are shown in Figure 1. The model reached an excellent data fit (χ^2^ = 788.07, *df* = 390, RMSEA = 0.050, CFI = 0.99, and TLI = 0.98). The results indicated that authoritarian leadership had significant and negative effects on teachers’ job satisfaction (β = -0.20, *p* < 0.01) and trust in the principal (β = -0.13, *p* < 0.01). Benevolence leadership had a significant effect on trust in the principal (β = 0.33, *p* < 0.05). Moral leadership had significant positive effects on trust in the principal (β = 0.47, *p* < 0.01), job satisfaction (β = 0.37, *p* < 0.05), and teacher commitment to students (β = 0.61, *p* < 0.01). Trust in the principal had significant effects on both job satisfaction (β = 0.34, *p* < 0.01) and teacher commitment to students (β = 0.41, *p* < 0.01). However, three paths were insignificant, namely, the effects of authoritarian leadership on teacher commitment to students and the effects of benevolence leadership on both job satisfaction and teacher commitment to students.

### Mediation Analysis

The mediating effects were further examined based on 5000 bootstrapping analysis. Hayes (2009) suggests that the indirect effect is significant if zero is not between the lower and upper bound in the 95% confidence interval. The results are shown in Table 2. Trust in the principal significantly mediated the effects of AL and ML on JS. In addition, trust in the principal also significantly mediated the effects of AL and ML on teacher commitment to students. For the effects of BL on job satisfaction and teacher commitment to students, the mediating effects were not significant. Therefore, H6 is partially supported. Table 3 summarizes the results of the hypothesis tests.

**TABLE 2:** Mediation analysis of trust in the principal on the effects of PL on teachers’ job satisfaction and commitment to students.

**Dependent variable**	**Independent variable**	**Direct effects**	**Mediation analysis**				
			**Estimates**	**SE**	**Two-tailed *p* value**	**95% Bootstrap CI**	
						**Lower**	**Upper**
7-8 JS	AL	–0.20	–0.05	0.02	0.03	–0.08	–0.01
	BL	–0.03	0.11	0.09	0.19	–0.03	0.25
	ML	0.37	0.16	0.09	0.07	0.01	0.31
CS	AL	–0.04	–0.06	0.03	0.04	–0.10	–0.01
	BL	–0.14	0.13	0.12	0.25	–0.06	0.33
	ML	0.61	0.19	0.10	0.06	0.02	0.36

**TABLE 3:** Summary of the results of the hypothesis tests.

**Hypotheses**	**Results**	**Remarks**
**H1**		
Negative relationship between AL and TJS and TCS	Supported	
**H2**		
Positive relationship between BL and TJS and TCS	Supported	
**H3**		
Positive relationship between ML and TJS and TCS	Supported	
**H4**		
Significant relationship between AL/BL/ML and TIP	Supported	
**H5**		
Positive relationship between TIP and TJS and TCS	Supported	
**H6**		
TIP as a mediator between AL, BL, ML, and TJS, TCS	Partially supported	A non-significant mediating effect of BL on JS and TCS via TIP

## Discussion

There has been increased interest in research on PL both in China (Farh and Cheng, 2000; Cheng et al., 2002; Wu et al., 2012; Zhang et al., 2015) and in international contexts (Aycan, 2006; Pellegrini and Scandura, 2008; Mansur et al., 2017). Following the suggestions proposed by meta-analysis on PL (Pellegrini and Scandura, 2008; Bedi, 2019), the present quantitative study examined the relationship between PL and teachers’ job satisfaction and commitment to students in the school context, with a particular focus on the mediating role of trust in the principal in mainland China.

### Effects of PL in Chinese Schools

The findings of this study contribute to the literature by providing empirical results of PL in Chinese schools. First, Chinese teachers tended to feel more unsatisfied with their job when principals showed more authoritarian leadership behaviors. The results are different with these studies conducted in companies, which revealed the positive links between AL and employees’ outcomes (Schaubroeck et al., 2017; Tian and Sanchez, 2017; Wang and Guan, 2018). The results are consistent with those of previous educational studies that found that authoritarian leadership is clearly and consistently negatively related to subordinate attitudes, behaviors, and performance (Cheng et al., 2002; Farh et al., 2008; Zheng et al., 2020).

Second, principal moral leadership was found to have a significant positive effect on teachers’ job satisfaction and commitment to students. This finding is congruent with previous educational studies that indicate the positive effect of moral leadership on teachers’ outcomes (Cheng et al., 2002; Farh et al., 2008). Furthermore, compared with the beta weight of the three dimensions of PL, the result lends credence to the finding of Farh et al. (2006) study, which argues that moral leadership always has the largest effect on teachers’ outcomes.

Third, the effects of the principal’s benevolent leadership on teachers’ job satisfaction and commitment to students were not significant. These results were inconsistent with previous studies in business contexts (Wu et al., 2012; Bedi, 2019). One possible reason may be the measurement. Items of benevolence leadership are mainly concerned with leaders’ caring about teachers’ personal lives, such as “My principal expresses concern about my daily life.” The principal’s concern and encouragement about teachers may not be directly associated with teaching or student issues; thus, there would be no significant relationship between benevolence leadership behaviors and teaching satisfaction and commitment to students. Another reason may be that school contexts are unlike business contexts (Wong, 2003). Chinese ideology deeply emphasizes human relationships (Farh and Cheng, 2000; Walker and Qian, 2018b), and it is likely that teachers “will perceive maintaining interpersonal harmony as part of their duty” (Chen et al., 2014, p. 811); thus, a higher level of benevolence leadership is unnecessary in bringing higher levels of satisfaction and commitment.

Finally, the three dimensions of principals’ PL were found to have different effects on trust in the principal. Specifically, benevolent and moral leadership had a significant positive effect on trust in the principal, whereas authoritarian leadership had a negative effect on trust in the principal. These results echo those of previous organizational studies, which showed that trust in a leader is significantly and positively associated with the leader’s benevolent and moral leadership but negatively related to their authoritarian leadership (Wu et al., 2012; Rawat and Lyndon, 2016). The results also confirm some researchers’ argument that creating a climate of trust in school is crucial for successful leadership practice (Bryk and Schneider, 2002; Day et al., 2011). The results provide more details regarding how principals can exert their influence on trust quality, i.e., moral and benevolent behaviors tend to enhance the quality of trust, while authoritarian behaviors may impair trust relationships.

### The Role of Trust in the Principal

The mediation results showed that trust in the principal had a positive effect on teachers’ job satisfaction and commitment to students. These findings are similar with those of previous studies that showed a positive relationship between trust in a leader and employee job satisfaction and commitment (Dirks and Ferrin, 2002; Skaalvik and Skaalvik, 2011; Van Maele and Van Houtte, 2012; Miao et al., 2013). It demonstrates that the quality of the teacher–principal social relationship informs the level of job satisfaction and commitment to student.

The mediation analysis further indicated that authoritarian and moral leadership had indirect effects on teachers’ job satisfaction and commitment to students and that the influence was mediated by trust in the principal. Therefore, H6 was partially supported. As expected, trust in the principal negatively mediated the effects of authoritarian leadership on teacher’s job satisfaction and commitment to students. A leader’s authoritarian leadership behaviors toward their employees may arouse negative reciprocation and thus break their trustworthiness in the eyes of their subordinates (Wu et al., 2012). Moral leadership could have a positive effect on teachers’ job satisfaction and commitment to students through the quality of trust in the principal, which is a finding that corroborates Day et al. (2011) argument that leaders enhance trust to bring positive organization outcomes.

The unexpected finding was that of the insignificant mediating effect of trust in the principal between benevolent leadership and teacher job satisfaction and commitment to students. These results were similar to Wu et al. (2012) findings that trust in supervisors does not mediate the relationship between benevolent leadership and subordinates’ in role/extra-role performance. A plausible explanation may be related to other psychological mechanisms, such as gratitude and repayment (Farh and Cheng, 2000; Wu et al., 2012). Based on the Chinese rule of reciprocity, benevolence may bring a high level of gratitude, but does not necessarily result in higher job satisfaction or commitment to students.

## Conclusion and Implications

The field of school leadership was dominated by leadership styles that originated in Anglo-American contexts such as instructional leadership and transformational leadership (Hallinger, 2011; Walker and Qian, 2018a). The study focuses on PL, a context-specific leadership style in Chinese contexts. It explores the influences of PL on teachers and how it operates in schools with empirical evidence. The results showed that authoritarian leadership had negative effects on teachers’ job satisfaction and trust in the principal, while moral leadership had positive effects on teachers’ job satisfaction/commitment to students and trust in the principal. The relationship between authoritarian leadership and teacher’s job satisfaction and commitment to students was negatively mediated by trust in the principal, whereas trust in the principal positively mediated the effects of moral leadership and teacher’s job satisfaction/commitment to students. The findings have some implications for improving principal leadership practices, especially in collective and hierarchical societies where paternalism is prevalent (Aycan, 2006; Pellegrini and Scandura, 2008; Hofstede et al., 2010; Mansur et al., 2017).

First, different dimensions of PL result in distinct organizational outcomes. As Mansur et al. (2017) suggested, it is necessary to “systematically consider the possibility that combinations of the PL dimensions may differ meaningfully in different contexts” (p. 710). The current results showed that the principal’s authoritarian leadership has a negative effect on teachers’ job satisfaction and trust in the principal. Along with the influence of rapid economic growth and social transformation, great changes in social culture and people’s traditional concepts have taken place (Wu et al., 2012). The desire for fairness has become a common pursuit of modern people. In this sense, authoritarian leadership that connotes total control and command over the teachers may be detrimental for teacher development and should be reduced as much as possible. Hence, the calls for undertaking actions such as empowering teachers, providing professional autonomy to teachers, and involving teachers in the decision-making process should come into the considerations of principals (Zheng et al., 2019).

Second, the study found that principal moral leadership has a positive effect on teacher job satisfaction, commitment to students, and trust in the principal. These findings may shed light on how principals could adopt appropriate leadership behaviors to enhance teachers’ work attitudes. In the Chinese culture, a good leader is always a role model. As Walker and Qian (2018b, pp. 7–8) argued, “aligned with traditional Confucian expectations of high levels of leader morality, school leaders in China are expected to be role models in various ways.” Personal traits such as selflessness, modesty, and honesty matter. Meanwhile, it is also critical for principals to lead teachers morally, such as by treating people fairly, by taking responsibility on the job and setting an example in all aspects.

In addition, trust relationships between principals and teachers could be enhanced to facilitate leadership practices. The current results confirmed the argument that maintaining trustful and harmonious relationships is considered fundamental for a better working environment, which will improve teachers’ performance (Hallinger, 2011; Yin and Zheng, 2018; Walker and Qian, 2018b). Trust in the principal was also found to positively mediate the effects of principal moral leadership on teachers’ job satisfaction and commitment to students. Hence, principals are suggested to undertake actions that promote the quality of trust relationships through their moral behaviors or interactions within their schools (Farh et al., 2008; Tschannen-Moran, 2014). For example, they could behave as trustworthy leaders who demonstrate the characteristics of honesty, openness, and reliability, who create more opportunities to interact with the teachers, and who are consistent in their words and actions (Bryk and Schneider, 2002; Tschannen-Moran, 2014). In summary, we agree with Farh et al. (2008) observation that most teachers expect their principals to be of high benevolence, high moral character, and low authoritarianism. Principals are suggested to “lead by winning subordinates’ respect and gratitude and rarely resort to positional authority” (Farh et al., 2008, p. 186).

When interpreting our findings, some limitations should be noted. First, the limited sample size did not employ a nationally representative sample of schools, which may restrain us from generalizing the results of our study to all schools in China. Thus, future research could include a larger sample size, involve teachers from different subjects, grade levels, schools, and regions. Second, we only used a questionnaire approach to gather the data of both independent and dependent variables from the teachers at the same time and at the same place, which may result in the issue of same source bias or common method variance. Thus, casual conclusions cannot be drawn from this cross-sectional study. Future research could consider a longitudinal study, and qualitative or mixed-method design could be conducted in future studies.

## Data Availability Statement

The raw data supporting the conclusions of this article will be made available by the authors, without undue reservation.

## Ethics Statement

The studies involving human participants were reviewed and approved by Southwest University Survey Research Ethics Committee. The patients/participants provided their written informed consent to participate in this study.

## Author Contributions

XS collected the data and drafted the first draft of the manuscript. XZ designed the research and revised the draft. ZY helped in data collection and finalized the manuscript. All authors contributed to the article and approved the submitted version.

## Conflict of Interest

The authors declare that the research was conducted in the absence of any commercial or financial relationships that could be construed as a potential conflict of interest.

## Appendix

### The Scales and Items Used in the Study

#### Paternalistic Leadership Scale (PLS)

Principal in this school:

1. Appears to be intimidating in front of teachers.
2. Brings me a lot of pressure when we work together.
3. Very strict with teachers.
4. Scolds me when I fail expected target.
5. Discipline me for violation of his/her principles.
6. Often shows his/her concern about me.
7. Understands my preference enough to accommodate my personal requests.
8. Encourages me when I encounter difficulties in work.
9. Would try to understand the real cause of my unsatisfied performance.
10. Trains and coached me when I lack required abilities at work.
11. Is responsible on the job.
12. Takes responsibility on job and never shirks his/her duty.
13. Sets an example to me in all aspects.
14. Well self-disciplined before demanding upon others.
15. Leads, rather than follows, teachers to deal with difficult tasks.

#### Trust in the Principal Scale (TiPS)

1. When teachers are struggling, our principal provides support for them.
2. Our principal ensures that all students get high quality teachers.
3. If my principal promised to do something, s/he would follow through.
4. In general, I believe my principal’s motives and intentions are good.
5. I feel free to discuss work problems with my principal without fear of having it used against me later.

#### Teacher Job Satisfaction Scale (JSS)

1. In most ways, being a teacher is close to my ideal.
2. My conditions of being a teacher are excellent.
3. I am satisfied with being a teacher.
4. So far I have gotten the important things I want to be a teacher.
5. If I could choose my career over, I would change almost nothing.

#### Teacher Commitment to Students Scale (TCSS)

1. It is my responsibility to advance all my students for high academic achievements.
2. All students can succeed and it is my mission to ensure their success.
3. It is my responsibility to ensure good social relations among my students.
4. I believe that being an educator makes me responsible for my students’ integration in the classroom.
5. I have to be aware of the social relations among students in my class and assist whenever needed to improve them.
